# Impact of high-iodine concentration contrast material for dual-energy CT angiography on arterial visualization: A single-blind, randomized controlled trial

**DOI:** 10.1016/j.ejro.2026.100733

**Published:** 2026-01-27

**Authors:** Yoshifumi Noda, Shoma Nagata, Taketo Suto, Masashi Asano, Takuma Ishihara, Takeshi Iwata, Toshiharu Miyoshi, Nobuyuki Kawai, Tetsuro Kaga, Masayuki Matsuo

**Affiliations:** a Department of Radiology, Gifu University, 1-1 Yanagido, Gifu 501-1194, Japan; b Department of Radiology, Massachusetts General Hospital, Harvard Medical School, 55 Fruit Street, White 270, Boston, MA 02114, USA; c Innovative and Clinical Research Promotion Center, Gifu University Hospital, 1-1 Yanagido, Gifu 501-1194, Japan; d Department of Radiology Services, Gifu University Hospital, Gifu 501-1194, Japan; e Department of Frontier Science for Imaging, Gifu University, 1-1 Yanagido, Gifu 501-1194, Japan; f Innovation Research Center for Quantum Medicine, Graduate School of Medicine, Gifu University, Gifu 501-1194, Japan; g Center for One Medicine Innovative Translational Research (COMIT), Institute for Advanced Study, Gifu University, 1-1 Yanagido, Gifu 501-1194, Japan

**Keywords:** Computed tomography angiography, Contrast media, Iodine

## Abstract

**Purpose:**

To investigate the effect of high-iodine concentration contrast material for dual-energy CT angiography (DECTA) on arterial visualizations by comparing it with medium-iodine concentration.

**Method:**

This prospective, single-blind, randomized controlled trial included 100 consecutive participants undergoing DECTA from November 2023 to February 2025. The participants were randomly assigned into two protocols: receiving high-iodine concentration contrast material of 370 mgI/mL (Group A, *n* = 51) and receiving medium-iodine concentration of 300 mgI/mL (Group B, *n* = 49). The axial, coronal, and volume-rendered (VR) images were reconstructed at 40 keV in both groups. Two radiologists reviewed three image types and assessed the arterial visualizations using a five-point scale. The primary outcome was the score for the iliolumbar artery on the VR images, whereas secondary outcomes were the scores for all others. The Wilcoxon rank sum test was conducted to compare the outcomes between the two groups.

**Results:**

No statistical significance in terms of the score for the iliolumbar artery on the VR images was found between the two groups; however, the median score was higher in Group A than in Group B (3.5 vs. 3.0; *P* = .05). The scores for secondary outcomes in Group A were equal to or greater than that in Group B, and significant differences were observed, especially in the small arteries, including the bronchial, internal thoracic, intercostal, left gastric, and inferior phrenic arteries (*P* < .05).

**Conclusion:**

In small arteries, the protocol with high-iodine concentration contrast material exhibited better arterial visualizations compared with medium-iodine concentration in DECTA at 40 keV.

## Highlights

• Although low-energy dual-energy CT angiography enables iodine reduction, benefit of high-iodine contrast remains debatable.
• Small arteries (bronchial, intercostal, left gastric, and inferior phrenic) were better visualized with 370 mgI/mL than in300 mgI/mL.
• At 40 keV dual-energy CT angiography, 370 mgI/mL provides better small-arterial visualization than 300 mgI/mL.

## 1 Introduction

CT angiography (CTA) has been widely used as a non-invasive tool for assessing aortic vascular anatomy and pathology [1], diagnosing aortic diseases [2], preoperative planning and follow-up of aortic aneurysm or valve disease [3], [4], and specifying feeding arteries of the tumor or related arteries to active bleeding [5], [6], [7], [8]. The use of dual-energy CTA (DECTA) has been recently reported. In particular, virtual monoenergetic images (VMIs) at low-energy levels (40–50 keV) generated from a dual-energy CT acquisition enable iodine load reduction [9], [10], [11], [12]. In these studies, low- or medium-iodine concentration contrast materials (240–320 mgI/mL) were used to reduce the iodine load and to earn the capacity load. For example, CTA for surveillance after endovascular aortic aneurysm repair is routinely recommended for detecting postoperative complications, such as endoleak [13], and these patients are often elderly and have relatively severe renal dysfunction. Therefore, we can agree that iodine reduction makes sense. However, the necessity of iodine reduction in all cases of DECTA at low-energy levels remains questionable.

While DECTA images at 40 keV in dual-source dual-energy CT have been reported to provide excellent visualization of small arteries [14], several drawbacks have also been reported, although these depend on the CT scanner model. Compared with single-energy CTA, DECTA demonstrated the degradation of arterial visualizations in intrapelvic small arteries [15]. Further, in recent years, deep-learning image reconstruction (DLIR) has been used more and more because of the increased image noise when using low-energy levels such as 40 keV [10], [16]. However, a previous study revealed that DECTA images at 40 keV reconstructed with DLIR demonstrated significantly inferior arterial visualization in small arteries compared with images reconstructed with hybrid-iterative reconstruction [10].

Generally, higher contrast enhancement can be achieved with a higher iodine delivery rate and a higher iodine concentration contrast material [17]. Indeed, high-iodine concentration contrast material yielded significantly higher attenuation in the aorta and coronary arteries [18]. Thus, we hypothesized that the use of high-iodine concentration contrast material may eliminate the previously described disadvantages. In particular, we wondered if high-iodine concentration contrast material could improve the visibility of small arteries, especially in the pelvis, in younger patients, etc., where contrast reduction is not mandatory. Therefore, this study aimed to investigate the effect of high-iodine concentration contrast material for DECTA at 40 keV reconstructed with DLIR on arterial visualizations by comparing them with those having medium-iodine concentration contrast material.

## 2 Materials and methods

### 2.1 Study participants and design

The Clinical Research Review Board of Nagoya University (CRB4180004) reviewed and approved this prospective, single-blind, randomized controlled trial. This study was registered in the Japan Registry of Clinical Trials (under the identifier jRCTs041230098). Data generated by the authors or analyzed during the study are available at Gifu University.

This study was designed as a randomized controlled trial to investigate the impact of high-iodine concentration contrast material for DECTA at 40 keV on arterial visualizations compared with medium-iodine concentration contrast material. The trial consecutively included participants aged ≥ 18 years, who were scheduled for CTA, and who signed written informed consent from November 2023 to February 2025. The exclusion criteria were 1) body weight of > 90 kg, 2) history of hypersensitive reaction to contrast material, 3) pregnant woman, 4) estimated glomerular filtration rate of < 30 mL/min/1.73 m^2^, and 5) loss of data.

### 2.2 Randomization and masking

Participants were randomly assigned (1:1) to receive one of two protocols: high-iodine concentration contrast material of 370 mgI/mL (Group A, iopromide of 370 mgI/mL; Bayer Yakuhin, Ltd.) or medium-iodine concentration contrast material of 300 mgI/mL (Group B, iopromide of 300 mgI/mL; Bayer Yakuhin, Ltd.) using a Research Electronic Data Capture system (REDCap) [19], stratified according to body mass index (< 25 kg/m^2^ and ≥ 25 kg/m^2^) and sex. Two or four permutation blocks were used for randomization. The reviewers conducted a single-blind assessment of arterial visualization under conditions where the iodine concentrations of each participant were not identifiable. Data were collected and managed using REDCap, hosted at Gifu University Hospital [19].

### 2.3 Primary and secondary outcomes

The primary outcome included the visualization of the iliolumbar artery on three-dimensional volume-rendered (VR) images. This is because it represents one of the arteries and reconstruction methods with the poorest visibility [12]. Secondary outcomes were visualizations of other arteries on axial, multiplanar reformatted (MPR), and VR images, overall image quality, and quantitative measurements, including CT attenuations of arteries, background noise, and signal-to-noise ratios (SNRs).

### 2.4 DECTA and contrast material injection

All examinations were performed with a rapid kilovoltage-switching dual-energy CT scanner (Revolution Apex Elite; GE HealthCare). Participants were scanned for CTA in the supine position craniocaudally, from the supraclavicular fossa to the pubic symphysis. The DECTA imaging parameters were tube voltage of 80/140 kilovolt peak (kVp), noise index of 8.5 HU at 5-mm slice collimation, variable tube current (GSI Assist; GE HealthCare), detector configuration of 128 detectors with 0.625-mm section thickness, beam collimation of 80 mm; rotation time of 0.5 s, pitch of 0.508:1, scan field-of-view of large body, and display field-of-view of 36 cm.

In both Groups A and B, the contrast material was intravenously injected at a rate of 4 mL/s using a commercially available power injector. A circle with a diameter of 15–20 mm was drawn to denote a region of interest (ROI) in the descending aorta at the bronchial carina level. Real-time fluoroscopic monitoring scans (140 kVp, 10 mA) were conducted 5 s after contrast material injection. The contrast material injection was discontinued when the bolus-tracking program SmartPrep (GE HealthCare) detected contrast enhancement reaching 40 HU [12] and was followed by a 20-mL saline injection at a 4 mL/s rate. CTA scanning was performed with an additional delay of 5 s after detecting threshold attenuation with the bolus-tracking program.

The CT dose-index volume (CTDI_vol_) and dose-length product (DLP) from the dose report were recorded.

### 2.5 Image reconstruction

The raw projection of the DECTA data at 1.25-mm section thickness with 50 % overlap was reconstructed at 40 keV using a medium-strength DLIR (TrueFidelity DL™; GE HealthCare). The reconstructed axial DECTA images were reformatted to MPR and three-dimensional VR DECTA images at a workstation (Advantage Windows 4.7; GE HealthCare). MPR images were reconstructed in the coronal plane with a 2.5-mm section thickness and no intersectional gap. The VR reconstruction parameters included a linear threshold with a lower threshold value of 125 HU set at 0 % opacity (completely transparent) and an upper threshold value of 600 HU set at 100 % opacity (completely opaque). The z-axis was selected for rotating the three-dimensional observation at every 10° over 360° of rotation, yielding 36 VR images.

### 2.6 Quantitative image analysis

A radiologist (M.A., with 2 years of experience in radiology), who was blinded to contrast material iodine concentration, measured the CT attenuations of the ascending aorta, aortic arch, thoracic aorta, upper abdominal aorta, lower abdominal aorta, and bilateral common iliac arteries on axial images using a circular ROI on a commercially available Digital Imaging software and Communications in Medicine viewer. Care was taken not to include the vessel wall, calcification, thrombus, medical devices, and artifacts in the ROI. The averaged CT attenuations of the ascending aorta, aortic arch, and thoracic aorta, that of the upper abdominal and lower abdominal aorta, and that of the bilateral common iliac arteries were determined as CT attenuations of the thoracic, abdominal, and pelvic arteries, respectively.

One standard deviation of CT attenuations of the homogeneous subcutaneous fat tissue at the level of the carina, upper pole of the left kidney, and cranial aspect of the femoral head, representing the thoracic, abdominal, and pelvic regions, was determined as the background noise for each participant. The SNRs were calculated by dividing the CT attenuation of the thoracic, abdominal, and pelvic arteries by the background noise at the corresponding anatomic regions.

### 2.7 Qualitative image analysis

Two experienced radiologists (S.N. and T.S., with 8 and 4 years of experience in radiology, respectively), who were blinded to contrast material iodine concentration, independently reviewed the axial, MPR, and VR DECTA images and then reached a consensus. The preset window settings of the axial and MPR images were initially fixed using an optimal window level of 140 HU and a window width of 680 HU [20], but the radiologists could adjust the window settings at their discretion during the evaluations.

The radiologists graded the arterial visualizations of the brachiocephalic, common carotid, subclavian, bronchial, internal thoracic, intercostal, common hepatic, proper hepatic, splenic, left gastric, gastroduodenal, inferior phrenic, superior mesenteric, inferior mesenteric, renal, lumbar, external iliac, internal iliac, iliolumbar, superior gluteal, inferior gluteal, obturator, and inferior epigastric arteries on the axial, MPR, and VR images, and overall image quality using a five-point scale. Arteries that are not detected by radiologists due to surgery and/or coil embolization were scored “Not applicable.” When a disagreement occurred, a consensus was reached by discussion. Supplementary Table 1 summarizes the detailed descriptions of the qualitative image analysis.

### 2.8 Statistical analysis

This study was designed to detect a superiority of at least 0.7 in the score for the iliolumbar artery on VR images between Groups A and B. The score for the medium-iodine concentration contrast material was 2.36 on average, with a standard deviation of 1.18, based on pre-study data from our institution. Using a Mann–Whitney *U* test, a simulation indicated that 100 participants were required to achieve 80 % power and a 5 % significance level.

The full analysis set (FAS) was defined as the population of randomly assigned cases, excluding those for which all measurements were not obtained. In the primary analysis, the arterial visualization scores of the iliolumbar artery were compared between Groups A and B using VR images, with the FAS as the target population, using the Mann–Whitney *U* test. In the secondary analysis, scores for other regions were compared using axial, coronal, and VR images, employing the same test as in the primary analysis. CT attenuations of arteries, background noise, SNR, and overall image quality were summarized and compared using the Mann–Whitney *U* test. The degree of agreement between the two reviewers’ scores was presented by Cohen’s Kappa coefficient. The characteristics of the participants are shown as the median and interquartile range (IQR) for the continuous variables and the frequency and percentage for the categorical variables. A two-sided *P*-value of < .05 indicated significance. All statistical analyses were conducted using R 4.4.1 (The R Project for Statistical Computing).

## 3 Results

### 3.1 Participants’ demographics

**Fig. 1:**
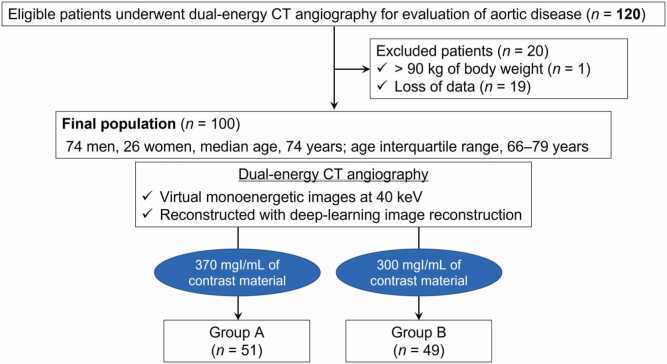
The flowchart illustrates the number of included participants. The participants were randomized into two groups: high-iodine concentration contrast material (370 mgI/mL; Group A) and medium-iodine concentration contrast material (300 mgI/mL; Group B).

A total of 120 participants were initially included; of these, 20 were excluded because of body weight of > 90 kg (*n* = 1) and loss of data (*n* = 19). The final sample consisted of 100 participants (74 men and 26 women; median age: 74 years [IQR: 66–79 years]; median body weight: 59 kg [IQR: 53–69 kg]; median body mass index: 23 kg/m^2^ [IQR, 20–25 kg/m^2^]) (Fig. 1). We found no difference in participants’ sex (*P* > .99), age (*P* = .29), height (*P* = .47), body weight (*P* = .49), and body mass index (*P* = .62) between Groups A and B (Table 1). Table 1 summarizes the clinical diagnoses. The median contrast volume was comparable between the two groups (*P* = .77); however, the iodine dose was greater in Group A than in Group B (35 g vs. 29 g; *P* < .001). We found no difference in CTDI_vol_ (*P* = .55) and DLP (*P* = .33) between Groups A and B.

**Table 1:** Participants’ demographics.

Variable	Overall	Group A	Group B	*P* value
Number of participants	100	51	49	
Men:Women	74:26	38:13	36:13	> .99
Age (y)	74 (66–79)	75 (65–84)	73 (66–78)	.29
Height (cm)	163 (158–170)	163 (157–170)	164 (159–171)	.47
Body weight (kg)	59 (53–69)	59 (53–69)	62 (53–68)	.49
Body mass index (kg/m^2^)	23 (20–25)	23 (21–25)	23 (20–25)	.62
Clinical diagnosis*				
Abdominal aortic aneurysm after EVAR	18 (18 %)	9 (18 %)	9 (18 %)	> .99
Thoracic aortic aneurysm after TEVAR	5 (5 %)	2 (4 %)	3 (6 %)	.68
Abdominal aortic aneurysm	41 (41 %)	22 (43 %)	19 (40 %)	.69
Others	37 (37 %)	18 (35 %)	19 (39 %)	.84
Contrast volume (mL)	95 (92–96)	95 (92–96)	95 (91–96)	.77
Iodine weight (g)	29 (28–35)	35 (34–36)	29 (27–29)	< .001
CT dose-index volume (mGy)	10 (8–10)	10 (8–10)	10 (8–10)	.55
Dose-length product (mGy*cm)	728 (614–813)	699 (608–799)	788 (624–843)	.33

Note.– EVAR = endovascular aortic aneurysm repair, TEVAR = thoracic endovascular aortic aneurysm repair.

Data are medians with interquartile ranges in parentheses.

*P* values were derived using Mann–Whitney *U* test or Fisher’s test.

*One case in Group B corresponds to both abdominal aortic aneurysm after EVAR and thoracic aortic aneurysm after TEVAR.

### 3.2 Quantitative image analysis

Table 2 summarizes the CT attenuation of the arteries, background noise, and SNRs. The median CT attenuation of all arteries was higher in Group A than in Group B (*P* < .001 for all). We found no difference in the median background noises of the thoracic (*P* = .24), abdominal (*P* = .91), and pelvic (*P* = .32) regions between the two groups. The median SNRs of all regions were higher in Group A than in Group B (*P* < .001 for all).

**Table 2:** CT attenuation of arteries, background noise, and signal-to-noise ratio.

Anatomic site	Group A	Group B	*P* value
CT attenuation (HU)			
Ascending aorta	1508 (1342–1878)	1241 (1104–1395)	< .001
Aortic arch	1564 (1336–1869)	1197 (1077–1358)	< .001
Thoracic aorta	1578 (1341–1909)	1231 (1106–1369)	< .001
Thoracic arteries (averaged)	1566 (1334–1864)	1218 (1081–1393)	< .001
Upper abdominal aorta	1554 (1332–1860)	1226 (1081–1383)	< .001
Lower abdominal aorta	1486 (1308–1853)	1217 (1068–1345)	< .001
Abdominal arteries (averaged)	1528 (1330–1851)	1219 (1073–1378)	< .001
Right common iliac artery	1429 (1188–1677)	1153 (1049–1288)	< .001
Left common iliac artery	1392 (1177–1610)	1169 (1029–1281)	< .001
Pelvic arteries (averaged)	1417 (1191–1693)	1158 (1038–1276)	< .001
Background noise (HU)			
Thoracic region	17 (15–20)	18 (16–21)	.24
Abdominal region	19 (17–20)	19 (17–21)	.91
Pelvic region	22 (20–24)	23 (20–26)	.32
Signal-to-noise ratio			
Thoracic region	90 (71–115)	69 (55–82)	< .001
Abdominal region	80 (69–95)	62 (54–75)	< .001
Pelvic region	65 (51–74)	53 (44, 57)	< .001

Note.– Data are medians with interquartile ranges in parentheses.

*P* values were derived using Mann–Whitney *U* test.

### 3.3 Qualitative image analysis

**Fig. 2:**
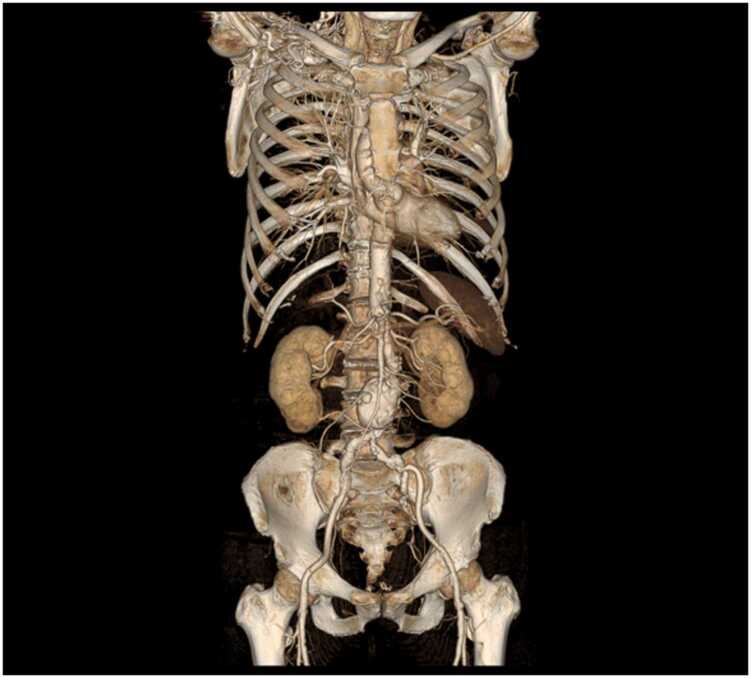

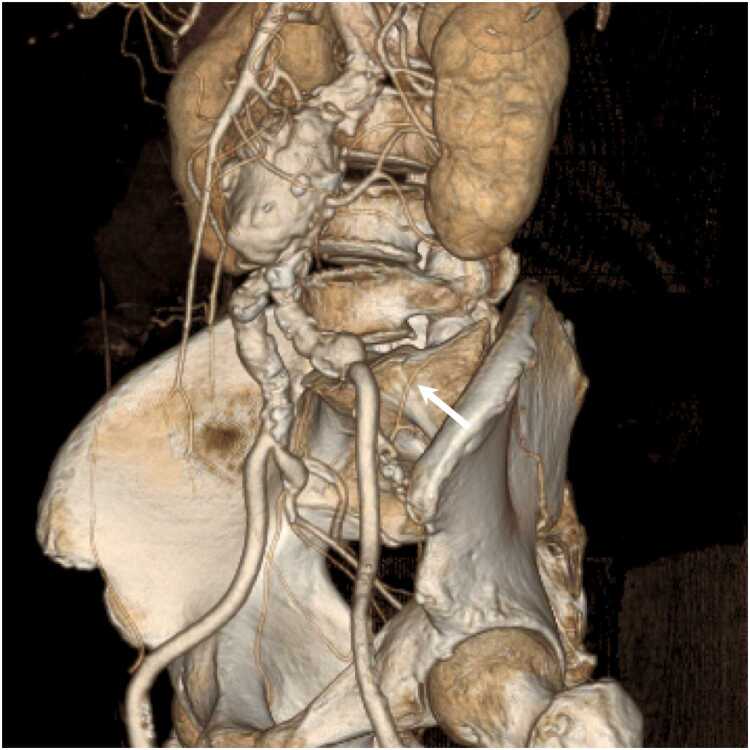
A 71-year-old man with abdominal aortic aneurysm. Dual-energy CT angiography obtained with a high-iodine concentration contrast material (370 mgI/mL). (a) Anterior whole-body volume-rendered (VR) image clearly illustrates the aorta and arterial branches. (b) VR image of the pelvis clearly shows the iliolumbar artery (arrow).

**Fig. 3:**
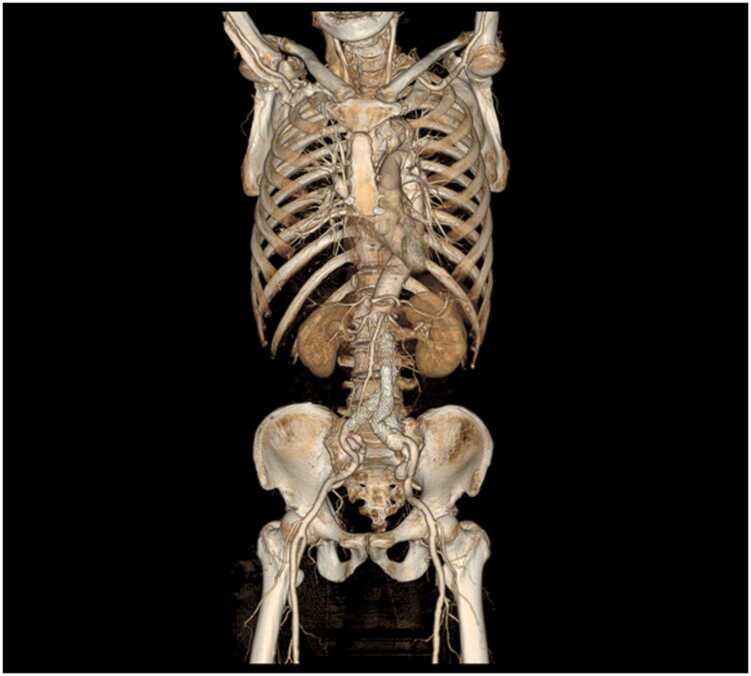

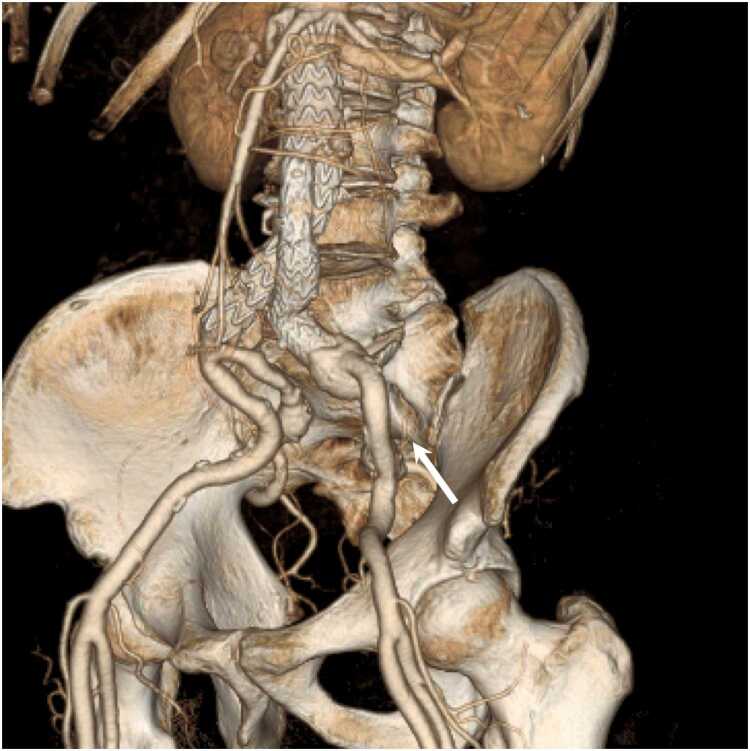
An 87-year-old man with abdominal aortic aneurysm after EVAR. Dual-energy CT angiography obtained with medium-iodine concentration contrast material (300 mgI/mL). (a) Anterior whole-body volume-rendered (VR) image clearly shows the aorta and arterial branches. (b) VR image of the pelvis illustrates the iliolumbar artery (arrow), but it is lighter than that in Fig. 2 and difficult to follow peripherally.

Table 3, Table 4, Table 5 summarize the arterial visualizations on the VR, axial, and MPR images. For the primary outcome, we found no difference in the arterial visualization of the iliolumbar artery on the VR images between the two groups; however, the median score was higher in Group A than in Group B (3.5 vs. 3.0; *P* = .05) (Fig. 2, Fig. 3). The following describes the secondary outcomes. For the VR images, the arterial visualizations of the internal thoracic (*P* = .03), intercostal (*P* < .001), left gastric (*P* = .01), inferior phrenic (*P* < .001), and superior mesenteric (*P* = .01) arteries were better in Group A than in Group B. We revealed no difference in other arteries (*P* = .12–.99). The *ĸ* values ranged from 0.47 to 1.00, indicating moderate to almost perfect agreement between the two reviewers. For axial images, the arterial visualizations of the bronchial (*P* = .04), intercostal (*P* = .04), and inferior phrenic (*P* = .04) arteries were better in Group A than in Group B. We found no difference in the other arteries (*P* = .10–>.99). The *ĸ* values ranged from 0.52 to 1.00, indicating moderate to almost perfect agreement between the two reviewers. For the MPR images, the arterial visualizations of the bronchial (*P* = .03), intercostal (*P* < .001), left gastric (*P* = .01), and inferior phrenic (*P* = .03) arteries were better in Group A than in Group B. We found no difference in the other arteries (*P* = .08–>.99). The *ĸ* values ranged from 0.51 to 1.00, indicating moderate to almost perfect agreement between the two reviewers.

**Table 3:** Arterial visualizations on volume-rendered images.

Arteries	Group A	Group B	*P* value	*ĸ* value (95 % CI)
Primary outcome				
Iliolumbar	3.5 (3.0–5.0)	3.0 (2.0–4.0)	.05	0.72 (0.62–0.83)
Secondary outcome				
Brachiocephalic	5.0 (5.0–5.0)	5.0 (5.0–5.0)	.99	1.00 (0.80–1.00)
Common carotid	5.0 (5.0–5.0)	5.0 (5.0–5.0)	.99	1.00 (0.80–1.00)
Subclavian	5.0 (5.0–5.0)	5.0 (5.0–5.0)	.99	1.00 (0.80–1.00)
Bronchial	1.0 (1.0–2.5)	1.0 (1.0–1.0)	.12	0.75 (0.61–0.88)
Internal thoracic	5.0 (4.0–5.0)	4.0 (3.0–5.0)	.03	0.60 (0.48–0.73)
Intercostal	4.0 (3.0–5.0)	2.0 (2.0–3.0)	< .001	0.61 (0.49–0.72)
Common hepatic	5.0 (5.0–5.0)	5.0 (5.0–5.0)	.60	1.00 (0.84–1.00)
Proper hepatic	5.0 (5.0–5.0)	5.0 (5.0–5.0)	.70	1.00 (0.85–1.00)
Splenic	5.0 (5.0–5.0)	5.0 (5.0–5.0)	.45	0.79 (0.65–0.93)
Left gastric	4.0 (3.0–4.0)	3.0 (2.0–4.0)	.01	0.61 (0.50–0.72)
Gastroduodenal	5.0 (5.0–5.0)	5.0 (5.0–5.0)	.78	0.89 (0.76–1.00)
Inferior phrenic	3.0 (2.0–3.0)	2.0 (1.0–2.0)	< .001	0.74 (0.62–0.85)
Superior mesenteric	5.0 (4.0–5.0)	4.0 (4.0–5.0)	.01	0.71 (0.56–0.86)
Inferior mesenteric	4.0 (3.0–4.0)	3.0 (3.0–4.0)	.34	0.78 (0.64–0.91)
Renal	5.0 (5.0–5.0)	5.0 (5.0–5.0)	.64	1.00 (0.86–1.00)
Lumbar	5.0 (4.0–5.0)	5.0 (4.0–5.0)	.33	0.47 (0.33–0.62)
External iliac	5.0 (5.0–5.0)	5.0 (5.0–5.0)	.40	0.86 (0.73–0.99)
Internal iliac	5.0 (5.0–5.0)	5.0 (5.0–5.0)	.99	0.86 (0.73–0.99)
Superior gluteal	5.0 (5.0–5.0)	5.0 (4.0–5.0)	.12	0.78 (0.64–0.92)
Inferior gluteal	5.0 (5.0–5.0)	5.0 (4.0–5.0)	.19	0.77 (0.64–0.90)
Obturator	4.0 (3.0–5.0)	3.0 (3.0–5.0)	.53	0.72 (0.61–0.82)
Inferior epigastric	3.0 (3.0–5.0)	3.0 (3.0–4.0)	.59	0.66 (0.55–0.76)

Note.– CI = confidence interval.

Data are medians with interquartile ranges in parentheses.

*P* values were derived using Mann–Whitney *U* test.

**Table 4:** Arterial visualizations on axial images.

Arteries	Group A	Group B	*P* value	*ĸ* value (95 % CI)
Secondary outcome				
Brachiocephalic	5.0 (5.0–5.0)	5.0 (5.0–5.0)	N.C.	N.C.
Common carotid	5.0 (5.0–5.0)	5.0 (5.0–5.0)	N.C.	N.C.
Subclavian	5.0 (5.0–5.0)	5.0 (5.0–5.0)	N.C.	N.C.
Bronchial	5.0 (4.0–5.0)	5.0 (3.0–5.0)	.04	0.77 (0.64–0.90)
Internal thoracic	5.0 (5.0–5.0)	5.0 (5.0–5.0)	.54	0.66 (0.46–0.85)
Intercostal	5.0 (5.0–5.0)	5.0 (5.0–5.0)	.04	0.60 (0.41–0.80)
Common hepatic	5.0 (5.0–5.0)	5.0 (5.0–5.0)	.99	1.00 (0.84–1.00)
Proper hepatic	5.0 (5.0–5.0)	5.0 (5.0–5.0)	> .99	1.00 (0.79–1.00)
Splenic	5.0 (5.0–5.0)	5.0 (5.0–5.0)	.55	1.00 (0.84–1.00)
Left gastric	5.0 (4.0–5.0)	5.0 (4.0–5.0)	.66	0.70 (0.55–0.84)
Gastroduodenal	5.0 (5.0–5.0)	5.0 (5.0–5.0)	.75	0.52 (0.35–0.70)
Inferior phrenic	5.0 (4.0–5.0)	5.0 (4.0–5.0)	.04	0.63 (0.49–0.77)
Superior mesenteric	5.0 (5.0–5.0)	5.0 (5.0–5.0)	.22	0.85 (0.70–1.00)
Inferior mesenteric	5.0 (4.0–5.0)	5.0 (5.0–5.0)	.64	0.75 (0.57–0.93)
Renal	5.0 (5.0–5.0)	5.0 (5.0–5.0)	.54	0.79 (0.60–0.99)
Lumbar	5.0 (5.0–5.0)	5.0 (5.0–5.0)	.97	0.66 (0.46–0.86)
External iliac	5.0 (5.0–5.0)	5.0 (5.0–5.0)	.33	1.00 (0.85–1.00)
Internal iliac	5.0 (5.0–5.0)	5.0 (5.0–5.0)	N.C.	1.00 (0.80–1.00)
Iliolumbar	5.0 (5.0–5.0)	5.0 (5.0–5.0)	.35	0.55 (0.40–0.70)
Superior gluteal	5.0 (5.0–5.0)	5.0 (5.0–5.0)	.71	0.72 (0.56–0.87)
Inferior gluteal	5.0 (5.0–5.0)	5.0 (5.0–5.0)	.95	0.74 (0.59–0.89)
Obturator	5.0 (5.0–5.0)	5.0 (5.0–5.0)	.10	0.66 (0.52–0.80)
Inferior epigastric	5.0 (4.0–5.0)	5.0 (4.0–5.0)	.64	0.64 (0.50–0.78)

Note.– N.C. = not calculated. The values could not be calculated because the two reviewers recorded the same scores for all cases.

Data are medians with interquartile ranges in parentheses.

*P* values were derived using Mann–Whitney *U* test.

**Table 5:** Arterial visualizations on multiplanar reformatted images.

Arteries	Group A	Group B	*P* value	*ĸ* value (95 % CI)
Secondary outcome				
Brachiocephalic	5.0 (5.0–5.0)	5.0 (5.0–5.0)	N.C.	N.C.
Common carotid	5.0 (5.0–5.0)	5.0 (5.0–5.0)	N.C.	N.C.
Subclavian	5.0 (5.0–5.0)	5.0 (5.0–5.0)	N.C.	N.C.
Bronchial	4.0 (3.0–5.0)	3.0 (2.0–4.0)	.03	0.80 (0.68–0.91)
Internal thoracic	5.0 (5.0–5.0)	5.0 (5.0–5.0)	.66	0.65 (0.45–0.84)
Intercostal	5.0 (5.0–5.0)	4.0 (4.0–5.0)	< .001	0.63 (0.47–0.79)
Common hepatic	5.0 (5.0–5.0)	5.0 (5.0–5.0)	> .99	1.00 (0.80–1.00)
Proper hepatic	5.0 (5.0–5.0)	5.0 (5.0–5.0)	> .99	1.00 (0.83–1.00)
Splenic	5.0 (5.0–5.0)	5.0 (5.0–5.0)	.56	0.85 (0.69–1.00)
Left gastric	5.0 (4.0–5.0)	4.0 (3.0–5.0)	.01	0.68 (0.54–0.81)
Gastroduodenal	5.0 (5.0–5.0)	5.0 (5.0–5.0)	.41	0.62 (0.46–0.78)
Inferior phrenic	4.0 (3.0–5.0)	3.0 (3.0–4.0)	.03	0.60 (0.47–0.72)
Superior mesenteric	5.0 (5.0–5.0)	5.0 (5.0–5.0)	.65	0.92 (0.78–1.00)
Inferior mesenteric	5.0 (4.0–5.0)	5.0 (4.0–5.0)	.47	0.72 (0.55–0.89)
Renal	5.0 (5.0–5.0)	5.0 (5.0–5.0)	.56	0.80 (0.63–0.97)
Lumbar	5.0 (5.0–5.0)	5.0 (5.0–5.0)	.44	0.51 (0.34–0.68)
External iliac	5.0 (5.0–5.0)	5.0 (5.0–5.0)	.33	1.00 (0.85–1.00)
Internal iliac	5.0 (5.0–5.0)	5.0 (5.0–5.0)	N.C.	1.00 (0.80–1.00)
Iliolumbar	5.0 (4.0–5.0)	5.0 (5.0–5.0)	.26	0.57 (0.42–0.72)
Superior gluteal	5.0 (5.0–5.0)	5.0 (5.0–5.0)	.61	0.74 (0.60–0.89)
Inferior gluteal	5.0 (5.0–5.0)	5.0 (5.0–5.0)	.35	0.89 (0.74–1.00)
Obturator	5.0 (4.0–5.0)	5.0 (4.0–5.0)	.70	0.74 (0.60–0.89)
Inferior epigastric	4.0 (3.0–5.0)	5.0 (4.0–5.0)	.08	0.66 (0.52–0.79)

Note.– N.C. = not calculated. The values could not be calculated because the two reviewers recorded the same scores for all cases.

Data are medians with interquartile ranges in parentheses.

*P* values were derived using Mann–Whitney *U* test.

We found no difference in the overall image quality in axial (Group A, 4.0 [IQR: 4.0–5.0] and Group B, 4.0 [IQR: 4.0–5.0]; *P* = .43), MPR (Group A, 4.0 [IQR: 4.0–4.0] and Group B, 4.0 [IQR: 4.0–4.0]; *P* = .82), and VR (Group A, 4.0 [IQR: 4.0–5.0] and Group B, 4.0 [IQR: 4.0–4.0]; *P* = .10) images among the two groups. The *ĸ* values ranged from 0.66 to 0.69, indicating substantial agreement between the two reviewers.

## 4 Discussion

Most of the studies on dual-energy CT angiography (DECTA) at low-energy level used low- or medium-iodine concentration contrast materials to reduce iodine load, and discussion of high-iodine concentration contrast material remains lacking. In this study, we compared the arterial visualizations in DECTA at 40 keV between the two protocols with medium- (300 mgI/mL) and high-iodine (370 mgI/mL) concentration contrast materials. We revealed better arterial visualizations in the protocol with a high-iodine concentration contrast material than in that with a medium-iodine concentration contrast material, especially in small arteries.

Virtual monoenergetic images at low-energy levels exhibit increased CT attenuations due to the approximation of the energy level closer to the k-edge of iodine at 33 keV [21]. Therefore, dual-energy CT enables the reduction of the iodine load and DECTA at low-energy levels (40–60 keV) with a reduced iodine dose (24 g), providing up to 185 % higher CT attenuation compared with single-energy CTA at 120 kVp with a standard iodine dose (33.3 g) [22]. The median iodine weight in Group A was 35 g in the present study, which is higher compared with previous studies investigating DECTA with reduced iodine (13–19 g) [9], [10], [11], [12]. This is a reasonable result because we investigated the usefulness of high-iodine concentration contrast material and is comparable with the amount of iodine in single-energy CTA [22] and abdominal contrast-enhanced dual-energy CT [23]. Thus, the use of high-iodine concentration contrast material did not cause an unacceptable increase in iodine load, and there are benefits from it, as our results indicate. In particular, trauma and postpartum hemorrhage require selective arterial embolization, which warrants a detailed understanding of the vascular anatomy, including the collateral circulation [24], [25]. Therefore, selecting a protocol that provides sufficient iodine loading and good visualization of the small arteries may be necessary in some cases.

Arterial enhancement and background noise may affect the arterial visualizations. Group A in this study demonstrated CT attenuations of approximately 1400–1500 HU for large arteries, which is approximately twice the CT attenuations of previous studies using 240 mgI/mL of contrast material [10], [11]. Moreover, as Noda et al. [12] discussed, the use of a newer reconstruction technique can improve arterial visualization. We applied deep-learning image reconstruction to this study, and the background noise was lower than that using hybrid-iterative reconstruction [11]. Reduced background noise may improve arterial visualization, especially in small arteries.

The present study defined the visualization of the iliolumbar artery on volume-rendered (VR) images as a primary outcome because its visualization was not good in our experience. No statistically significant difference was found between the two groups; however, Group A demonstrated better visibility of the iliolumbar artery on VR images than Group B. A previous study investigating DECTA at 40 keV with 240 mgI/mL of contrast material gave the iliolumbar artery on VR images a score of 2.4 using the same assessment method as ours [10]. Using this study as a comparison, a score of 3.0 in Group B using 300 mgI/mL and a score of 3.5 in Group A utilizing 370 mgI/mL indicate that increasing the iodine concentration of contrast material improves small arterial visualization. The improved visualizations of all small arteries, not just the iliolumbar artery, on the VR images demonstrate the usefulness of high-iodine concentration contrast material.

Our study had several limitations. First, the median body weight of the participants in our study was 59 kg. This body weight distribution may be lower than that of the Western population. Further, participants with > 90 kg of body weight were excluded from this study because of the significant image quality degradation experienced with the dual-energy CT scan. The present results cannot be directly extrapolated to Western populations. This limitation may also partly explain why no statistically significant difference was observed in the primary endpoint. High-iodine-concentration contrast material may be particularly beneficial in patients with a larger body habitus, and we believe that further evaluation is warranted. Second, only one radiologist performed CT attenuation measurements. Finally, we only used rapid kilovoltage-switching dual-energy CT scanners from a single vendor. Given the growing number of studies demonstrating the excellent visualization of very small vessels with photon-counting CT [26], [27], [28], [29], it is clear that further improvements can be achieved with photon-counting CT.

In conclusion, we revealed that arterial visualizations were better in the protocol with high-iodine concentration contrast material (370 mgI/mL) than with medium-iodine concentration contrast material (300 mgI/mL) in dual-energy CT angiography (DECTA) at 40 keV, especially for small arteries. However, it should be noted that a high-iodine protocol is not necessary for the general purpose of DECTA when evaluating the great arteries. It may be helpful in selected cases evaluating small arteries. This knowledge enables the creation of tailored DECTA protocols based on the patient’s renal function and urgency.

## CRediT authorship contribution statement

**Takuma Ishihara:** Methodology. **Toshiharu Miyoshi:** Conceptualization. **Takeshi Iwata:** Investigation. **Tetsuro Kaga:** Data curation. **Nobuyuki Kawai:** Data curation. **Masayuki Matsuo:** Writing – review & editing. **Shoma Nagata:** Data curation. **Yoshifumi Noda:** Writing – original draft. **Masashi Asano:** Data curation. **Taketo Suto:** Data curation.

## Ethical statement

The Clinical Research Review Board of Nagoya University (CRB4180004) reviewed and approved this prospective, single-blind, randomized controlled trial. This study was registered in the Japan Registry of Clinical Trials (under the identifier jRCTs041230098). Informed consent was obtained from all participants.

## Funding

This study was supported by Bayer Yakuhin, Ltd.

## Declaration of Competing Interest

The authors declare that they have no known competing financial interests or personal relationships that could have appeared to influence the work reported in this paper.
