# Supplementary material for: Impact of high-iodine concentration contrast material for dual-energy CT angiography on arterial visualization: A single-blind, randomized controlled trial
Source: Eur J Radiol Open. 2026 Jan 27;16:100733. doi: 10.1016/j.ejro.2026.100733 (PMC12870465; doi:10.1016/j.ejro.2026.100733)
Supplement: Supplementary file 1 — Supplementary material [file mmc1.docx]

**Supplementary Table 1**. Rating Scale for Arterial Visualization and Overall Image Quality

| Image quality metric | Description of scale |
| --- | --- |
| Arterial visualization | 1. No vascular segments were clearly visualized |
|  | 1. Intermediate between grades 3 and 1 |
|  | 1. Indicated that nearly half of all vascular segments were clearly visualized |
|  | 1. Indicated intermediate between grades 5 and 3 |
|  | 1. All vascular segments from the trunk to the subsegmental peripheral artery were clearly visualized |
|  | N.A. No vessel to be evaluated |
| Overall image quality | 1. Unacceptable |
|  | 1. Suboptimal |
|  | 1. Acceptable |
|  | 1. Good |
|  | 1. Excellent |

Note.– N.A. = not applicable.
